# Failure of tDCS to modulate motor excitability and speech motor learning

**DOI:** 10.1016/j.neuropsychologia.2020.107568

**Published:** 2020-09

**Authors:** Charlotte E.E. Wiltshire, Kate E. Watkins

**Affiliations:** Wellcome Centre for Integrative Neuroimaging, Department of Experimental Psychology, Radcliffe Observatory Quarter, University of Oxford, OX2 6GG, UK

**Keywords:** Non-invasive brain stimulation, Speech production, Articulatory motor cortex, Electrical stimulation

## Abstract

Transcranial direct current stimulation (tDCS) modulates cortical excitability in a polarity-specific way and, when used in combination with a behavioural task, it can alter performance. TDCS has the potential, therefore, for use as an adjunct to therapies designed to treat disorders affecting speech, including, but not limited to acquired aphasias and developmental stuttering. For this reason, it is important to conduct studies evaluating its effectiveness and the parameters optimal for stimulation. Here, we aimed to evaluate the effects of bi-hemispheric tDCS over speech motor cortex on performance of a complex speech motor learning task, namely the repetition of tongue twisters. A previous study in older participants showed that tDCS could modulate performance on a similar task. To further understand the effects of tDCS, we also measured the excitability of the speech motor cortex before and after stimulation. Three groups of 20 healthy young controls received: (i) anodal tDCS to the left IFG/LipM1 and cathodal tDCS to the right hemisphere homologue; or (ii) cathodal tDCS over the left and anodal over the right; or (iii) sham stimulation. Participants heard and repeated novel tongue twisters and matched simple sentences before, during and 10 min after the stimulation. One mA tDCS was delivered concurrent with task performance for 13 min. Motor excitability was measured using transcranial magnetic stimulation to elicit motor-evoked potentials in the lip before and immediately after tDCS. The study was double-blind, randomized, and sham-controlled; the design and analysis were pre-registered. Performance on the task improved from baseline to after stimulation but was not significantly modulated by tDCS. Similarly, a small decrease in motor excitability was seen in all three stimulation groups but did not differ among them and was unrelated to task performance. Bayesian analyses provide substantial evidence in support of the null hypotheses in both cases, namely that tongue twister performance and motor excitability were not affected by tDCS. We discuss our findings in the context of the previous positive results for a similar task. We conclude that tDCS may be most effective when brain function is sub-optimal due to age-related declines or pathology. Further study is required to determine why tDCS failed to modulate excitability in the speech motor cortex in the expected ways.

## Highlights

• TDCS did not modulate performance on tongue twisters in healthy young adults.
• TDCS applied concurrently with task failed to modulate motor excitability.
• TDCS may be most effective in brains where brain function is sub-optimal.

## 1 Introduction

Transcranial direct current stimulation (tDCS) is a non-invasive brain stimulation technique that can modulate cortical excitability. tDCS exerts its effect by passing a weak electric current between two electrodes placed on the scalp. This current induces polarity-specific modulation such that the anode up-regulates, and the cathode down-regulates local cortical excitability (Bikson and Rahman, 2013; Nitsche and Paulus, 2000). The electrophysiological effects of tDCS over the motor cortex can be demonstrated by measuring changes in excitability via changes in the size of motor evoked potentials (MEPs) recorded from muscles of interest in response to single pulse transcranial magnetic stimulation (TMS) over the cortical representation (Stagg and Nitsche, 2011). Specifically, when targeting the representation of the hand in primary motor cortex (M1), the size of the MEP elicited by TMS in the contralateral hand muscles was increased by anodal tDCS (a-tDCS), and decreased by cathodal tDCS (c-tDCS) relative to sham stimulation (Nitsche et al., 2003; Stagg et al., 2009).

When used in combination with a behavioural task, a single session of tDCS can modulate performance. For speech production specifically, there is some evidence to suggest that tDCS can modulate performance in neurologically intact speakers (for example: Buchwald et al., 2019; Cattaneo et al., 2011; Deroche et al., 2017; Holland et al., 2011; Lametti et al., 2018). In order to realise the potential for tDCS as an effective tool for treatment or rehabilitation of speech disorders, it is important to evaluate the parameters optimal for stimulation. It is also important to use tasks that challenge the healthy speech motor system and are capable of eliciting errors commonly seen in populations with speech pathologies. One such task is the repetition of sentences with complex articulation such as tongue twisters (Wilshire, 1999). In the current study, we were interested in whether tDCS over the speech motor cortex could affect performance on a task requiring the repetition of novel tongue twisters.

Two previous studies evaluated the effects of tDCS on performance of tongue twisters in two separate populations of healthy adults. In the first (Fiori et al., 2014), three groups of healthy older adults (mean age = 57 years; N = 30, 10 per group) received a-tDCS or c-tDCS (both 2 mA; 20 min) or sham stimulation over left inferior frontal cortex while repeating familiar Italian tongue twisters. Participants' response times and accuracy in repeating tongue twisters were successfully modulated during stimulation: a-tDCS significantly increased accuracy and reduced response times relative to baseline measures, whereas c-tDCS significantly reduced accuracy and increased response times from baseline; sham stimulation had no effect. The second study (Wong et al., 2019) compared the effects of a-tDCS (2 mA for 20 min) over Broca's area and sham stimulation on reading of Cantonese tongue twisters in a younger population (mean age = 27 years; N = 30, 15 per group). Speech rate and accuracy improvements on the task were not modulated by a-tDCS, however. It should be noted that unlike the first study, the participants did not receive tDCS while they performed the task.

Here, we aimed to further evaluate the effects of tDCS on tongue twister repetition in healthy young adults by taking both a behavioural and electrophysiological measurement before and after tDCS paired with a tongue twister task. In the current study, we added electrophysiological measurements of motor excitability to assess tDCS changes using TMS-induced MEPs. This may be important in explaining individual differences in the anticipated modulatory effects of tDCS on behaviour. Furthermore, the ability to predict who may respond well to the tDCS would be important for the use of tDCS as a therapeutic tool in patients.

We made a number of changes to the previous experimental designs (Fiori et al., 2014; Wong et al., 2019) with the aim of optimizing the effects of stimulation and evaluating them with appropriate controls in a randomized double-blind sham-controlled study. The study design and analysis plan were pre-registered on the Open Science Framework (https://osf.io/p84ys/).

For the behavioural task, we added a control condition (repetition of simple sentences) to determine whether the anticipated effects on complex motor speech performance would generalise. The tongue twisters used were, by necessity English, and unfamiliar, unlike the previous studies, which used familiar Italian (Fiori et al., 2014) and novel Cantonese (Wong et al., 2019) ones. These unfamiliar sentences were also shorter than the Italian ones which were familiar and long (~20 syllables) because we were concerned about demands on working memory, whereas the Cantonese ones ranged from five to 67 syllables and were presented visually for reading instead. Following piloting, our participants were given 4.5 s to produce their responses, whereas the Italian study had intervals of 20 s.

For tDCS, we used a bi-hemispheric electrode montage rather than uni-hemispheric used previously and ensured the electrodes covered the lip representation of M1. This may have resulted in a slightly more posterior positioning of the large 5 × 7 cm electrodes relative to the previous studies, which centred the active electrode over left inferior frontal cortex and the return electrode over the supra-orbital ridge on the right. Bi-hemispheric montages have been shown to be at least as effective as uni-hemispheric ones (Fiori et al., 2017; Meinzer et al., 2014; Prichard et al., 2014) or can even improve the effects on task performance (Drummond et al., 2017; Vines et al., 2008; Waters et al., 2017). Of the few studies that directly compared uni- and bi-hemispheric montages, one reported greater improvement in motor learning after bi-lateral tDCS (1 mA, 20 min) compared to uni-hemispheric tDCS (Naros et al., 2016), and another reported the opposite pattern of results on cortical excitability (1 mA, 5 min) (Nitsche and Paulus, 2000).

For tDCS, we chose parameters that would deliver an effective dose of tDCS whilst ensuring effective blinding of the participants. We used 1 mA tDCS compared with 2 mA used in the previous studies and our duration of stimulation was shorter (13 compared with 20 min). Identical stimulation parameters (1 mA for 13 min) were used to demonstrate increases in motor cortical excitability of 150% above baseline lasting for at least 90 min after the end of the stimulation (Nitsche and Paulus, 2001). In our experience and that of other reports, blinding of the participant is not always achieved at 2 mA due to the increased somatosensory experiences, e.g. tingling and itching under the electrodes (O'Connell et al., 2012). In addition, many studies have demonstrated the effectiveness of 1 mA tDCS for similar time periods on behaviour (López-Alonso et al., 2015; Monte-Silva et al., 2013; Nitsche et al., 2008, 2003; Stagg and Nitsche, 2011; Strube et al., 2016).

In the current study, we used single pulses of transcranial magnetic stimulation applied over the representation of the lips in the left M1 to elicit MEPs in the contralateral orbicularis oris muscle. This allowed us to gain more insight into the cortical effects induced by tDCS alongside behavioural outcomes in the same individuals, thus providing more sensitive information about the individual variability of cortical responses to tDCS (Chew et al., 2015; Guerra et al., 2018). The degree to which cortical excitability changes (as indexed by changes in MEP size) could predict changes in behaviour is unknown.

## 2 Methodology

### 2.1 Sample size

We aimed to have sufficient power to detect an effect of tDCS on task performance of moderate size. In our view, a moderate effect size is the minimum needed if the combination of stimulation and task has potential for use therapeutically in patient studies. We determined that 20 participants per group (n = 60) were required based on a Cohen's d = 0.8 with 80% power at the standard 0.05 alpha error probability (for a directional *t*-test).

### 2.2 Participants

Seventy-one healthy participants were recruited. Participants self-reported that they were all right-handed, native English speakers with normal or corrected-to-normal vision and normal hearing. We were unable to record reliable MEPs from the lips in 11 participants most likely due to between-subject variance in the orientation of the lip representation on the anterior bank of the central sulcus. The remaining 60 participants were aged between 18 and 42 years (mean = 22.3, SD = 4.85); there were 30 men and 30 women.

The University of Oxford Central University Research Ethics Committee approved the study. Participants gave informed written consent to participate in the study, in accordance with the Declaration of Helsinki, and with the procedure approved by the committee.

### 2.3 Design

The study was a double-blind randomized controlled study. Block randomization (block size 6) was used to assign 60 participants to one of four stimulation configurations with an allocation ratio of 2:1 (1 mA tDCS with either anode on left or cathode on left, 20 participants each; sham with either anode on left or cathode on left, 10 participants each). Males and females were randomized separately to balance the groups. The researchers were blinded to the allocation of group by using the ‘study mode’ of the DC-stimulator (NeuroConn GMbH, Ilmenau, Germany). A member of the research group who was not involved in the study assigned a 5-digit code to each participant. The link between the code and the stimulation group was not revealed until all 60 complete data sets were collected.

### 2.4 Procedure

#### 2.4.1 Tongue twister task

36 novel tongue twisters (7 or 8 syllables) were taken from a previous published set (Fossett et al., 2016). For each tongue twister, a corresponding simple sentence was created that did not contain sequences of similar consonants. To achieve this, one word was retained from the tongue twister and all other words were replaced by a word with the same number of syllables and syntactic class but with different onsets. For example, “Chad bravely wore Anne's little shoes” was created to match the tongue twister “Brad bravely broke Brooke's brittle blades”. A female, native-English speaker was recorded speaking the tongue twisters and control sentences in a soundproof booth. Tongue twisters and simple sentences were matched for mean duration and amplitude. Recordings were presented via TMS-compatible insert earphones (ER.1 model from Etymotic, Elk Grove Village, IL, USA).

For each trial, participants were instructed to repeat the sentence immediately after the cessation of the audio recording within a four and a half second response window. The task comprised three blocks of 24 sentences (12 tongue twisters and 12 simple sentences, presented in a randomized order). After each block, participants took a 30s break. The duration of the task was 13 min. Participants practised the task on three simple sentences prior to the first run.

In pre-registering our analysis, it was necessary to choose a primary outcome measure. Response time was measured as the duration from the end of the auditory stimulus presentation to the end of the participant's spoken response. This measure was used previously (Fiori et al., 2014) and encompasses reaction time, speech rate, and is affected by response accuracy as it includes the cost in time of any hesitations, self-corrections or other types of error or dysfluency.

#### 2.4.2 tDCS stimulation

**Fig. 1:**
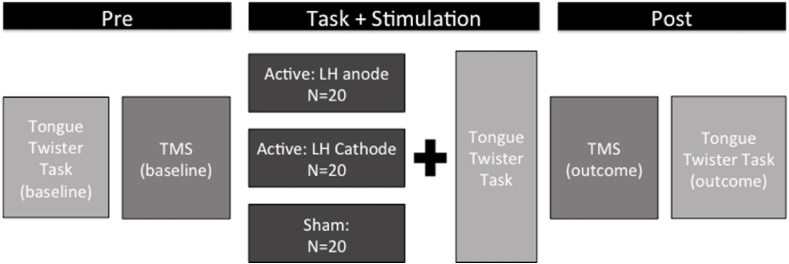
**Study Design**. The stimulation site and motor threshold were determined at baseline and 20 MEPs obtained. The tongue-twister task involved repetition of 36 tongue twisters and 36 simple sentences. The stimulation (anodal, cathodal and sham) was applied concurrently with the task. Twenty MEPs were obtained at the end of the stimulation period and the task was repeated again without stimulation.

1 mA of stimulation or sham was delivered using study mode on a neuroConn DC-STIMULATOR PLUS (neuroConn GMbH, Ilmenau, Germany) via two 5 × 7 cm saline-soaked electrodes. Two groups received active stimulation during which the intensity of current was ramped up slowly for 15 s before being held constant for 13 min and ramped down for 15 s. During the sham stimulation, the intensity of the current was ramped up slowly for 15 s before being held constant for 30 s and ramped down for 15 s. These sham stimulation parameters delivered current at an ineffective dosage (Jog et al., 2016) (see Fig. 1).

**Fig. 2:**
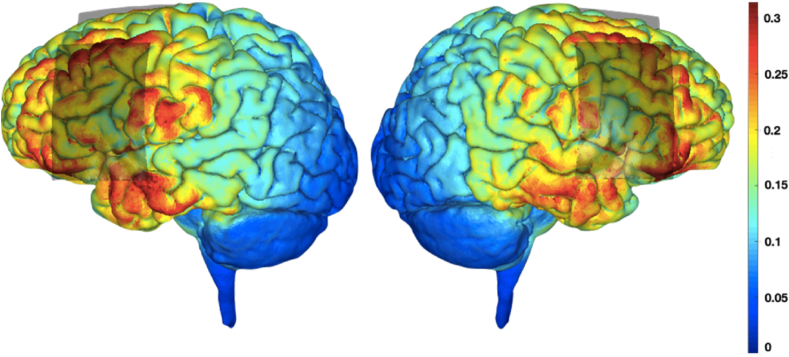
**Simulated current flow.** For this example, the anode is placed on the left hemisphere and the cathode on the right hemisphere. Red indicates high current density; blue indicates low current density. Simulation created using simnibs.com (Saturnino et al., 2015). (For interpretation of the references to colour in this figure legend, the reader is referred to the Web version of this article.)

The anodal group received bi-hemispheric, active stimulation with the anode placed on the left hemisphere IFG/M1 and the cathode placed over the homologous area in the right hemisphere. The cathodal group received bi-hemispheric, active stimulation with the reverse electrode configuration: the cathode over left hemisphere IFG/M1 and the anode over the homologous area in the right hemisphere. To ensure blinding of the researcher, placement of the electrodes was counterbalanced for the sham group such that half were placed in the anodal condition described above and half were placed in the cathodal configuration. A simulation of current density and flow based on the equipment and parameters used in this set up is illustrated in Fig. 2.

#### 2.4.3 TMS and electrophysiological recording

Single-pulse TMS was delivered over the left hemisphere lip representation of M1 using a DuoMag 200 stimulator through a 70-mm figure-eight coil. The coil was placed tangential to the skull, to induce a horizontal current flow from posterior to anterior under the junction of the two wings of the figure-eight coil. Surface electrodes (22 × 30 mm ABRO neonatal electrocardiogram electrodes) were attached to the right corner of the lower and upper lip (orbicularis oris muscle) in order to record the electrical activity of the underlying muscle. The ground electrode was attached to the forehead.

**Fig. 3:**
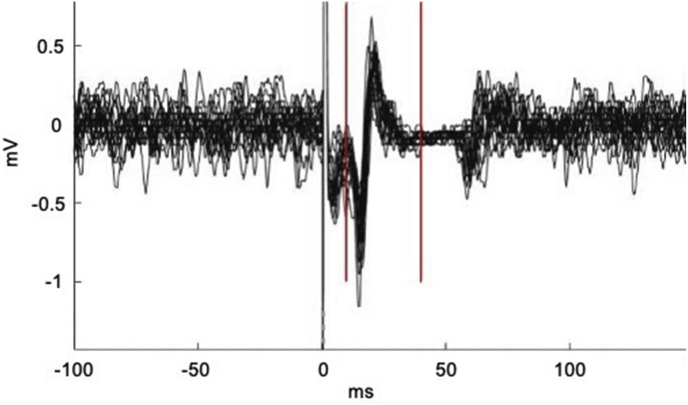
Example EMG recording of 20 MEPs overlaid. The peak-to-peak amplitude of the MEPs was calculated automatically within a window of 10–40 ms (represented by vertical red lines) after the TMS pulse was delivered (solid vertical black line). (For interpretation of the references to colour in this figure legend, the reader is referred to the Web version of this article.)

The lip representation of M1 was located by moving the coil first ventrally to a point one third of the distance between Cz and the tragus of the left ear and then slightly further ventrally and anteriorly until single TMS pulses elicited an MEP in the contralateral orbicularis oris muscle. The lip motor hotspot was then identified by moving the coil around this area until the largest and most reliable MEP was elicited. The stimulation threshold was identified as the stimulation intensity needed to achieve an average MEP size that was at least 1 mV peak-to-peak for 10 consecutive pulses whilst the participant maintained lip muscle contraction at approximately 20% of their maximum (referred to hereafter as the 1-mV threshold). The average percentage of maximum stimulator output needed to achieve the 1-mV threshold was 58.5% and did not differ among the three tDCS groups (see Table 1). Participants were trained to maintain 20% of their maximum contraction via visual feedback. Subsequently, 20 single pulses of stimulation were automatically delivered at random intervals of between 5 and 6.5 s at this threshold to the left lip motor cortex to elicit the MEPs for measurement. Participants were given visual feedback of the power of their contraction during the measurement period in order to maintain the contraction of the lip muscles. An example of 20 MEP recordings is shown in Fig. 3.

**Table 1:** Summary of participant demographics and results. Means are provided with range and SEM or SEM in parenthesis.

Stimulation Group	Anodal (N = 20)			Cathodal (N = 20)			Sham (N = 20)		
Age (years)	22.45 (19–33; 0.81)			23.25 (19–42; 1.59)			21.25 (18–29; 0.57)		
TMS output (% max output)	56.6 (37–66; 1.61)			57.9 (36–76; 2.22)			62.2 (46–80; 2.04)		

Time point (relative to tDCS)	Pre	During	Post	Pre	During	Post	Pre	During	Post

Tongue Twisters Response time (s)	3.25 (0.07)	3.14 (0.06)	3.08 (0.08)	3.22 (0.08)	3.01 (0.08)	2.92 (0.09)	3.13 (0.07)	3.00 (0.08)	2.93 (0.08)
Simple Sentences Response time (s)	2.89 (0.07)	2.77 (0.07)	2.79 (0.09)	2.80 (0.10)	2.63 (0.09)	2.56 (0.10)	2.75 (0.07)	2.61 (0.08)	2.62 (0.08)
Power of lip contraction (mV)	0.117 (0.008)	NA	0.116 (0.008)	0.142 (0.112)	NA	0.141 (0.011)	0.122 (0.010)	NA	0.120 (0.009)
MEP size (mA)	1.15 (0.05)	NA	1.05 (0.05)	1.16 (0.06)	NA	1.13 (0.07)	1.19 (0.07)	NA	1.14 (0.08)

tDCS = transcranial direct current stimulation, SEM = standard error of the mean, mV = millivolts, mA = milliamps, NA = Not applicable.

For the MEP measures at post-stimulation, 20 MEPs were elicited using the same threshold (% of stimulator output) and position. BrainSight neuronavigation equipment (Rogue Research Inc, Montreal, Quebec, Canada) was used to ensure the precise area (accurate to 2 mm) is stimulated in an identical way (position, orientation and tilt of the coil) before and after the tDCS stimulation.

The peak-to-peak amplitude of the MEPs was calculated automatically within a window of 10–40 ms after the TMS pulse (see Fig. 3). The average height of the rectified EMG signal for 200 ms before the TMS pulse was used to estimate the power of the contraction for each trial. As the amount of contraction is linearly related to the size of the MEP, we used analysis of covariance for each participant to adjust the MEP size for the amount of contraction (see Watkins et al., 2003). This can be done using a standard statistical package in which the mean MEP size is provided adjusted for the covariate (in this case, the amount of contraction) for each condition (in this case, measurements taken pre- and post-stimulation) for each subject analysed separately. The analysis is equivalent to using a linear regression between MEP size (y) and amount of contraction (x) to determine the slope (β) of the relationship and predict the adjusted MEP size according to the formula:

$$y_{adj\;i\;}=y_{raw\;i}-\;\beta \left(x_{raw\;i}-\;\bar{x}\right).$$

This adjusted MEP size was used in the analyses below.

## 3 Results

The results for each group are summarised in Table 1.

Raw data are available on OSF (https://osf.io/berp5/). The following conventional hypothesis testing analyses were unchanged from the pre-registration analysis plan, however where these analyses resulted in a non-significant effect, additional Bayesian analyses were included in order to compare the weight of evidence in support of the null hypothesis with that in support of the experimental hypothesis.

### 3.1 Control analyses

**Fig. 4:**
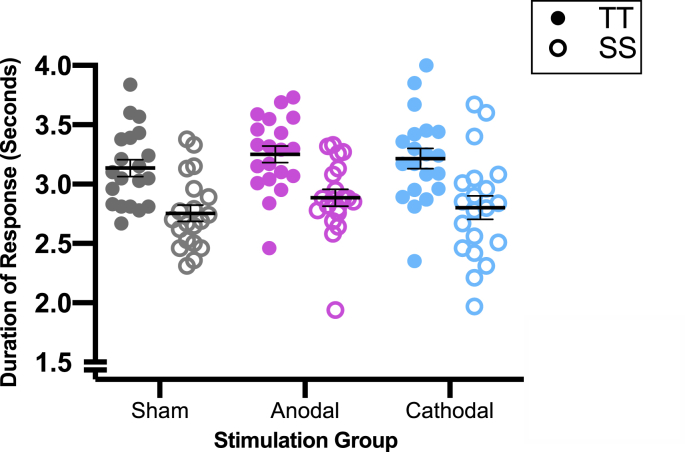
**Baseline Task Performance.** Duration of responses by sentence type (TT and SS) and stimulation group. Each point represents the mean of an individual participant. The horizontal black line is the group mean, error bars represent one SEM.

Firstly, the baseline data were analysed to confirm that the tongue twisters were repeated with longer durations compared with simple sentences (positive control) and also that there were no existing group differences at baseline. These data are plotted in Fig. 4.

For the measure of response duration obtained pre-stimulation in the three groups, a 2 × 3 repeated measures analysis of variance (RM ANOVA), with sentence type as a within-subject factor (TT vs SS) and stimulation group as between-subject factor (anodal vs. cathodal vs. sham). There was a significant main effect of sentence type (F (1,57) = 509.72, *p* < .001, d = 5.4) due to longer response times for TT compared with SS in all three groups. The main effect of group (F (2,57) = 0.65, *p* = .523) and the interaction between sentence type and group (F (2,57) = 0.69, *p* = .502) were not significant.

### 3.2 Q1. Behavioural: does anodal tDCS enhance learning to repeat tongue twisters in healthy young adults?

We used a 2 × 3 RM ANOVA with sentence type as a within-subject factor (TT, SS) and stimulation group as a between-subject factor (anodal, cathodal and sham) to test our hypotheses: H1A) people receiving anodal stimulation over the left hemisphere will show significantly greater improvements in sentence durations when repeating tongue twisters compared with people receiving cathodal or sham stimulation; H1B) people receiving cathodal stimulation over the left hemisphere will show significantly lower improvements in sentence durations when repeating tongue twisters compared with the people receiving sham stimulation; and H1C) the effect of anodal stimulation on sentence durations will be greater for repetition of tongue twisters compared with repetition of simple sentences. To assess learning, we analysed the dependent measure of change in duration over time (post- minus pre-stimulation). A significant main effect of sentence type showed the magnitude of reduction in response time was significantly greater for TT than for SS (F (1,57) = 9.67, *p* = .003, d = 0.82). The main effect of group (F (2,57) = 2.20, *p* = .120) and the interaction between group and sentence type (F (2,57) = 0.83, *p* = .92) were not significant.

Bayesian analyses were used to assess the weight of evidence in support of the null hypothesis (compared with the experimental hypothesis) for the main effect of group and the interaction between group and sentence type. We used the anovaBF function with default priors from the BayesFactor package in R (Morey and Rouder, 2018) for a Bayesian Type II ANOVA. The Bayes factor for the interaction between stimulation group and sentence type indicated that the data were approximately 7.14:1 times in favour of the null hypothesis (relative to the experimental hypothesis), which is considered a substantial effect (Jarosz and Wiley, 2014). The Bayes factor for the main effect of group, however, was 1.04:1 times in favour of the null hypothesis, which is inconclusive (note that this does not indicate support for either hypothesis). We therefore carried out pairwise comparisons for the tongue twister sentences only, using ttestBF from the BayesFactor package in R (Morey and Rouder, 2018). Directional tests were used as we predicted that the anodal group would have a greater improvement (reduction in response time) compared with the sham and cathodal groups. In contrast, we predicted that the cathodal group would show less improvement (longer response times) compared with the sham group. The Bayes factors indicated substantial evidence in favour of the null hypothesis for the comparison of anodal vs sham (BF = 4.56:1), cathodal vs sham (BF = 8.6:1), and anodal vs cathodal (BF 7.14:1) groups.

**Fig. 5:**
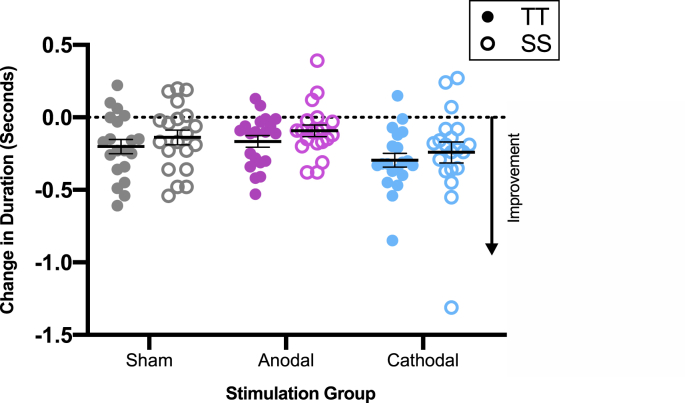
No Effect of tDCS on Learning to Repeat Tongue Twisters. Change in duration of responses (post- minus pre-stimulation) for tongue twisters (TT) and simple sentences (SS) by stimulation group. Each point represents the mean of an individual participant. Horizontal black line shows group mean. Error bars represent SEM.

All groups showed a significant reduction in the duration of responses for repetition of sentences (i.e. one-sample *t*-test against no change: tongue twisters t (59) = −8.16, *p* < .001, d = 1.05; simple sentences t (59) = −4.80, *p* < .001, d = 0.62). This effect was significantly greater for the tongue twisters compared with the simple sentences, as shown in Fig. 5 and confirmed by the main effect of sentence type above. However, the evidence in support of the null hypotheses for the interaction involving stimulation group and sentence type and the pairwise comparisons for group differences further support our conclusion that task performance was not modulated by the anodal (or the cathodal) stimulation as predicted.

### 3.3 Q2. Electrophysiological: does tDCS change excitability in the motor system underlying speech production?

**Fig. 6:**
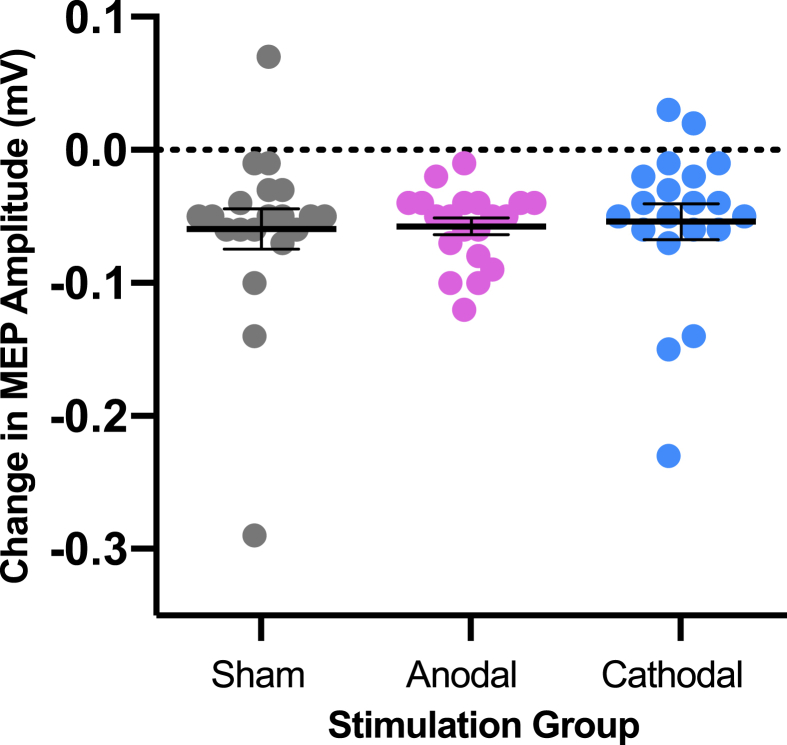
**No Effect of tDCS on Excitability in the Motor System Underlying Speech Production.** Change in MEP size (post- minus pre-stimulation) by stimulation group. Each point represents the mean of an individual participant. Horizontal black line shows group mean. Error bars represent SEM. Dashed line at y = 0 represents no change in MEPs.

To test our Hypothesis 2A (anodal tDCS over the left hemisphere will increase excitability in the speech motor system measured contralaterally compared with cathodal and sham stimulation), two t-tests compared change in MEP size (post- minus pre-stimulation) between the anodal and cathodal groups and separately between the anodal and sham groups. Directional t-tests were used as we expected an increase in MEP amplitude in the anodal group compared with the other two groups, which we expected to remain either unchanged (sham) or to decrease in amplitude (cathodal group). The change in MEP size for the anodal group was not significantly bigger than the changes for either of the other two groups (anodal vs sham: t (38) = 0.80, *p* = .469; anodal vs cathodal: t (38) = −0.31, *p* = .380), as shown in Fig. 6. Directional Bayesian t-tests were run to assess these null results. This revealed substantial evidence in favour of the null for the anodal vs sham comparison (BF = 3.01:1) and the anodal vs cathodal comparison (BF = 3.98:1).

To test our Hypothesis 2B (cathodal tDCS over the left hemisphere will decrease excitability in the speech motor system measured contra-laterally compared with sham stimulation) a single *t*-test was used to compared change in MEP size between cathodal and sham groups. A directional test was used as we expected a decrease in MEP amplitude in the cathodal group compared with the sham group, which we expected would not change. The change in MEP size for the cathodal group was not significantly different from that in the sham group (t (38) = 0.29, *p* = .387). A directional Bayesian *t*-test revealed substantial evidence in favour of the null hypothesis (BF = 3.92:1).

### 3.4 Q3. Does the change in motor excitability predict learning on the behavioural task?

To test our Hypothesis 3A that change in motor excitability will correlate positively with the size of the improvement on the tongue twister task we correlated the change in MEP size with the change in duration of repetition of tongue twisters. The change in MEP size did not correlate with change in duration of repetition of tongue twisters for any of the groups (Anodal: r = −.33, *p* = .161; Cathodal: r = −0.07, *p* = .757; Sham: r = −0.14, *p* = .517). We also planned to compare the slopes of the regression lines in the three groups separately. However, because none of the correlations were significant, these comparisons were not carried out.

### 3.5 Exploratory analysis

The following analyses were not planned.

#### 3.5.1 Did task performance change during stimulation?

We also tested whether tDCS affected task performance during stimulation as this time point was measured in the previous study that found evidence of a stimulation group effect (Fiori et al., 2014). The means and SEM for each group are shown in Table 1.

The change in duration of response times for tongue twister sentences from pre- to during-stimulation was significantly different from zero (no change) for all groups (all *p* < .005), however neither anodal nor cathodal were different from sham (anodal vs sham: t (38) = −0.64, *p* = .529; cathodal vs sham: t (38) = 1.09, *p* = .284). Bayesian t-tests show inconclusive support for the null hypothesis for both of the above comparisons (anodal vs sham BF = 2.77:1; cathodal vs sham BF = 2.04:1). Therefore, all groups improved their performance during the tDCS but the evidence to suggest that tDCS had no effect on this improvement is inconclusive.

#### 3.5.2 Did the amount of muscle contraction during MEP measurements differ pre- and post-tDCS?

Differences in the power of the lip muscle contraction during TMS affects MEP size. We therefore tested whether this had changed from pre- to post-tDCS in any of our groups (see Table 1). There were no differences between the power of contraction at pre- and post-tDCS for any of the stimulation groups (sham: t (19) = 0.47, *p* = .645; anodal: t (19) = 0.859, *p* = .401; cathodal: t (19) = 0.29, *p* = .777).

MEP amplitudes significantly decreased from pre- to post-stimulation for all of the groups (see Fig. 6) (one-sample *t*-test against no change, i.e. zero: t (59) = −8.24, *p* < .001, d = 1.06).

#### 3.5.3 Were participants blind to whether they were receiving real or sham stimulation?

In order to test whether participants could guess if they were receiving real or sham stimulation, a 2 × 2 Chi-square test was performed. Responses from seven participants were not recorded (sham = 3, real = 4). Of the 17 people who received sham, 8 guessed it was sham and 9 that it was real stimulation; of the 36 people who received real stimulation, 11 guessed it was sham and 25 that it was real. These proportions are not different from chance (χ^2^ (1, N = 53) = 1.37, *p* = .242) indicating that participants were successfully blinded to the type of stimulation they were receiving.

## 4 Discussion

We tested whether tDCS could modulate performance on a tongue-twister task in healthy young adults. Sixty participants received either sham (n = 20), or bi-hemispheric tDCS with the anode on the left (n = 20) or right (n = 20). Their ability to repeat sentences with either complex (tongue twisters) or simple articulation was tested before and after tDCS concurrent with the task. TDCS did not modulate performance on the task. Participants showed an improvement in performance and this was greater for the tongue twisters than for the simple sentences but these changes did not differ among the three groups. These results align with a recent study that also failed to show that tDCS over the left inferior frontal cortex modulated performance on a tongue twister task (Wong et al., 2019) but differ from a similar report that successfully modulated performance using tDCS, with anodal improving performance and cathodal worsening performance compared with sham stimulation (Fiori et al., 2014). The effects of tDCS on behaviour in neurotypical populations have often been difficult to replicate (Guerra et al., 2018; Tremblay et al., 2016b). This could in part reflect individual differences in the expected response to brain stimulation. Therefore, we also used TMS to elicit MEPs as a measure of motor excitability before and after the tDCS; we predicted that motor excitability would be modulated by tDCS in a polarity-specific way and that individual differences in the behavioural effects of tDCS might be explained by differences in the change in motor excitability. Unexpectedly, our results showed no modulatory effect of tDCS on motor excitability either. Participants showed a small reduction in excitability from pre- to post-stimulation but this did not differ among the three groups.

These results contrast with those of a study that successfully modulated behavioural performance using tDCS (Fiori et al., 2014) but are in accord with a failed replication attempt (Wong et al., 2019). There are a number of differences in the study protocols between the current study and those previously reported that might explain the differences in results. Some of these were already highlighted briefly in the introduction. We used bi-hemispheric rather than uni-hemispheric stimulation hoping to increase the effect on behaviour as shown previously for motor learning (Drummond et al., 2017; Naros et al., 2016; Vines et al., 2008; Waters et al., 2017). There is, however, a lack of evidence on the effect of using a bi-hemispheric stimulation on motor cortical excitability. It is possible that whilst bi-hemispheric stimulation is beneficial for behavioural outcomes, this relationship may not be true for measures of cortical excitability (Nitsche and Paulus, 2000; Tremblay et al., 2016a). Further replication of these effects alongside individualised current flow modelling using MRI is needed to understand whether this is the case. We also used a lower current amplitude of 1 mA rather than 2 mA in order to ensure that participants were blind to real vs. sham stimulation whilst not decreasing its effectiveness (Chesters et al., 2017; Ho et al., 2016). Our debriefing indicated that participants were successfully blinded to stimulation type at 1 mA. As noted in the introduction, we also reduced the duration of stimulation but we believe this was unlikely to explain our null results as numerous reports of tDCS applied to the motor cortex in healthy humans show behavioural modulation with stimulation durations of between 10 and 16 min (Monte-Silva et al., 2013; Nitsche et al., 2008, 2003; Stagg and Nitsche, 2011). In sum, we believe the changes to the stimulation protocol described above are unlikely explanations for our failure to detect a modulatory effect on task performance in this study. We turn next to the changes we made to the behavioural protocol.

Our study used different stimuli for the task and introduced a control task (repetition of simple sentences). Necessarily, the language of the sentences was changed from Italian to English. If the speech motor effect is generalisable across languages, this would not explain the difference in results. Our tongue twisters were novel (as in Wong et al., 2019) rather than well-known (as in Fiori et al., 2014) which may recruit different brain regions and is a potential explanation for the different findings. In addition, our sentences were shorter than those used previously; the mean response time for tongue twisters at baseline in our study was 3.2 s, whereas it was ~4.5 s in the previous study (Fiori et al., 2014). The inter-stimulus interval was also shorter (reduced from 20 to 4.5 s) compared with a previous study (Fiori et al., 2014). It cannot be ruled out that the introduction of more simple sentences combined with the reduction in duration of the tongue twisters reduced the effectiveness of the tDCS to modulate performance. Our study may have been less sensitive to changes in behaviour between stimulation groups because of these differences in stimuli.

The most important difference between our study and the previous study in which tDCS successfully modulated task performance was the age of the participants. Both studies included neurotypical participants but those in (Fiori et al., 2014) were considerably older (mean = 57 years; SD = 11) than those in the current study (mean = 22.3 years, SD = 4.8) and indeed in the other study that failed to find a significant effect of tDCS on tongue twister performance (Wong et al., 2019; mean age = 27 years, SD = 11.26). In our view, this age difference is the most plausible explanation for the different findings among the studies. For example, in a previous study, tDCS with a concurrent visuomotor adaptation task significantly improved performance of healthy older adults to the level of that seen in younger adults without stimulation (Panouillères et al., 2015) suggesting that age-related declines in task performance can be reversed using tDCS. Similarly, a study comparing bi-hemispheric and uni-hemispheric a-tDCS on a language learning task found age-dependent effects in that only the elderly group showed task improvement; performance in the group of young healthy adults was not modulated by either type of stimulation (Fiori et al., 2017). Taken together, the reduction in the sentence length for our study and our focus on younger healthy adults may have reduced our sensitivity to the modulatory effects of tDCS.

In the current study, we added electrophysiological measurements of motor excitability to assess tDCS changes using TMS-induced MEPs. Our aim was to explain individual differences in the anticipated modulatory effects of tDCS on task performance by variability in the modulatory effects of tDCS on motor excitability. In some respects, we succeeded, in that the failure to find an effect of tDCS on task performance was mirrored by a lack of effect of tDCS on motor excitability. Nevertheless, this result was unexpected given previous established results that tDCS modulates MEP size in a polarity-specific way (Nitsche and Paulus, 2000) and specifically the identical stimulation parameters used in one of our groups (1 mA of a-tDCS for 13 min over the motor cortex) increased excitability by 150% relative to baseline for at least 90 min after the stimulation ended (Nitsche and Paulus, 2001). It is important to note, however, that these modulatory effects were found in studies that did not involve performing a concurrent task (Nitsche and Paulus, 2001, 2000; Stagg and Nitsche, 2011). A previous report found that anodal tDCS (1 mA, 20 min) applied without a concurrent task increased MEP size as expected but when the stimulation was applied in combination with a digit-sequence task, MEP size was not modulated even though task performance measurably improved (Amadi et al., 2015). One plausible explanation of these results is that during task performance the brain alters its excitability to counteract the effects of tDCS. Such homeostatic regulation would therefore abolish the measurable effects of tDCS on motor excitability. This suggestion could be important in explaining the variable results within the tDCS literature and aid optimisation of tDCS protocols. It is also possible that the reduction in MEP size for all groups is representative of fatigue effects or changes in arousal. This would explain the changes in the sham group, where no modulation by tDCS was expected. Fatigue or reduced arousal effects on excitability could have masked the anticipated reduction in MEP size in the cathodal group. We would expect that the anticipated increase in excitability due to anodal stimulation to have at least counteracted these effects, however. In sum, we would still expect to see group differences on excitability even if this were reduced in general from pre- to post-stimulation. Further investigation into the modulation of the speech motor system by tDCS applied with or without performance of a concurrent task is required.

It is possible that tDCS is most effective when the area of cortex being stimulated functions atypically. For example, left ventral premotor cortex is known to be underactive during speaking in people who stutter compared with controls (Watkins et al., 2008) and anodal tDCS over this area improved fluency in people who stutter compared with sham stimulation (Chesters et al., 2018). Similarly, tDCS led to modulated performance on a digit sequence task in the non-dominant, but not the dominant hand of neurotypical adults (Boggio et al., 2006). In our opinion, the negative results for both task and motor excitability in the current study are best explained by the fact that our healthy young adults function optimally, which renders modulation by tDCS ineffective. Note, that this is not simply due to a behavioural ceiling effect as there was room for improvement on task performance both in terms of latency and accuracy, which would have affected response time. Furthermore, the cathodal stimulation was expected to lower performance and was also ineffective.

In summary, our study failed to demonstrate the previously reported polarity-specific modulatory effects of tDCS on speech motor control in a typical population. Our study had a sample size double that of the previous study and was sufficiently powered to detect a similarly sized effect. Bayesian analyses of our data also confirmed substantial evidence in support of the null hypothesis both for the effect of tDCS on task and on motor excitability. The factor of participant age and how this interacts with brain function is the most likely explanation for this failure to detect an effect should one exist. The alternative explanation is that the effect cannot be replicated but the changes we made to the protocol and the population difference in age precludes such a firm conclusion. The lack of modulation by tDCS on motor excitability is consistent with the lack of effect on behaviour but we believe this is better explained by homeostatic regulation of cortical excitability that may occur during task performed concurrently with tDCS in the typically functioning brain.

## CRediT authorship contribution statement

**Charlotte E.E. Wiltshire:** Conceptualization, Methodology, Formal analysis, Investigation, Writing - original draft, Writing - review & editing, Visualization, Project administration, Funding acquisition. **Kate E. Watkins:** Conceptualization, Methodology, Writing - original draft, Writing - review & editing, Supervision, Project administration, Resources, Funding acquisition.

## Declaration of competing interest

None.
